# Contrast-FEL—A Test for Differences in Selective Pressures at Individual Sites among Clades and Sets of Branches

**DOI:** 10.1093/molbev/msaa263

**Published:** 2020-10-16

**Authors:** Sergei L Kosakovsky Pond, Sadie R Wisotsky, Ananias Escalante, Brittany Rife Magalis, Steven Weaver

**Affiliations:** 1 Institute for Genomics and Evolutionary Medicine, Temple University, Philadelphia, PA; 2 Emerging Pathogens Institute, University of Florida, Gainesville, FL

**Keywords:** evolutionary model, codon model, selective differences, mechanisms adaptation

## Abstract

A number of evolutionary hypotheses can be tested by comparing selective pressures among sets of branches in a phylogenetic tree. When the question of interest is to identify specific sites within genes that may be evolving differently, a common approach is to perform separate analyses on subsets of sequences and compare parameter estimates in a post hoc fashion. This approach is statistically suboptimal and not always applicable. Here, we develop a simple extension of a popular fixed effects likelihood method in the context of codon-based evolutionary phylogenetic maximum likelihood testing, Contrast-FEL. It is suitable for identifying individual alignment sites where any among the K≥2 sets of branches in a phylogenetic tree have detectably different *ω* ratios, indicative of different selective regimes. Using extensive simulations, we show that Contrast-FEL delivers good power, exceeding 90% for sufficiently large differences, while maintaining tight control over false positive rates, when the model is correctly specified. We conclude by applying Contrast-FEL to data from five previously published studies spanning a diverse range of organisms and focusing on different evolutionary questions.

## Introduction

When the same gene is subjected to different selective environments, population processes, or exogenous adaptive forces in different sets of species or other taxonomic units (e.g., viral or bacterial isolates or cancer lineages), especially if it leads to functional adaptation or differentiation, we expect to find distinct molecular signatures of selection among these sets. At the nucleotide or protein level, this difference can manifest as variation in evolutionary rates across groups of species, for example, in the *rbcL* gene of monocots where species with shorter generation times showed higher evolutionary rates (Gaut et al. 1992), or both across sites and lineages, leading to *heterotachy*—a process that is widespread in protein evolution and has been studied extensively (Lopez et al. 2002; Whelan et al. 2011). At the codon level, a commonly adopted modeling framework is to allow the strength of selection, represented by the ratio of nonsynonymous (*β*) and synonymous (*α*) substitution rates, ω:=β/α, to vary across branches or both branches and sites. The primary focus of methodological development has been to estimate *ω* and compare it with the neutral expectation ω:=1 (e.g., see Delport et al. [2008] or Arenas [2015] for a review). Here, we focus instead on the methods for comparing *ω* across sets of branches; these methods are relatively few and far-between (cf. table 1). We further assume that the branches are partitioned into groups using additional sources of information, and not inferred as a part of the evolutionary analysis. Yang (1998) developed a likelihood-ratio test (LRT) to compare *gene-average* selective pressures among different sets of branches in the tree. By design, Yang’s method relies on pooling data across sites and branches and lacks resolution to identify individual sites subject to selective differentials. A more recent model allowing clade-level effects in a site-mixture framework can infer fractions of sites experiencing a clade-level shift but not individual sites that differ between branches (Baker et al. 2016). Another approach allows *ω* to vary across sites and branches as a random effect, with the group-level effect to distinguish the sets of branches (Wertheim et al. 2015). This method can answer more refined questions (e.g., is selection on one set of branches relaxed or intensified relative to the other set?), but it still lacks the site-level resolution. Several approaches have been specifically designed to detect evidence of directional selection toward a preferred subset of residues at specific sites and/or branches in a phylogenetic tree. Parto and Lartillot (2017) developed random effects mutation-selection models that allow selective profiles of amino acids to vary across sites and branches and can be fitted in a Bayesian phylogenetic framework; they can identify specific sites and specific residues subject to directional selection. Tamuri et al. (2012) implemented a conceptually similar model in a fixed effects model and fitted it using maximum likelihood approaches to identify which residues are preferred at specific sites. Murrell et al. (2012) augmented standard codon models to reward/penalize substitutions toward specific residues along predefined sets of branches at specific sites (using fixed effects) and applied it to the evolution of drug resistance (DR) in HIV. RifeMagalis et al. (2020) applied a version of Contrast-FEL to determine which HIV-1 envelope sites might be evolving differentially between three anatomical compartments in a single host. Recent work by Jones et al. (2020) develops a model where changes in selective pressures can be correlated with changes in phenotypes along clades or branches, fits a random effects model to determine whether a fraction of sites support such correlated evolution, and uses an empirical Bayes approach to recover individual sites where this may occur. Numerous methods have also been developed to contrast evolutionary patterns among continuous traits (e.g., Beaulieu et al. 2012), but they operate on conceptually different data (continuous characters) and are therefore not properly comparable.

**Table 1.** Methods for Comparing Selection among Different Sets of Branches.

Method	Application	Statistical Framework
Yang (1998)	Gene-wide differences in average *ω*	ML, LRT
Baker et al. (2016)	Gene-wide differences in distributions of *ω*	Random effects ML, LRT
Wertheim et al. (2015)	Gene-wide differences in distributions of *ω*	Random effects ML, LRT
Tamuri et al. (2012)	Site level amino acid preferences	Fixed effects ML
Murrell et al. (2012)	Site level directional selection	Fixed effects ML, LRT
Parto and Lartillot (2017)	Site and branch-level amino acid preferences	Bayesian MCMC mixture
	*ω* variation at site/branch level	
Jones et al. (2020)	Gene-level phenotype—selection correlation at a fraction of sites	Random effects ML, ancestral state sampling, LRT, empirical Bayes
Contrast-FEL	Site-level differences in *ω* between sets of branches	Fixed effects ML, LRT

Note.—ML, maximum likelihood; LRT, likelihood-ratio test; MCMC, Markov Chain Monte Carlo.

Here, we fully develop and validate a fixed effects site-level model (Contrast-FEL) and an LRT to formally test the hypothesis of differences in *ω* ratios between two or more groups of branches using an LRT. None of the existing methods, with the possible exception of Parto and Lartillot (2017) (a Bayesian approach designed to answer somewhat broader questions, whereas Contrast-FEL is a frequentist approach), can directly test for differences in *ω* between groups of branches at a specific site, providing the rationale for a new method in this application domain. The method Jones et al. (2020) has some advantages, for example, accounting in uncertainty in branch assignments, allowing for multinucleotide substitutions, and heterotachy, but lacks a direct test for differences at specific sites, instead relying on an empirical Bayes procedure, and does not account for site-to-site synonymous rate variation (Wisotsky et al. 2020); a direct comparison with this method is not straightforward. We evaluate Contrast-FEL using comprehensive simulations, and, having established reasonable statistical behavior even under a misspecified model simulation scenario, apply it to five disparate empirical data sets previously analyzed for differential selection among branch sets. Contrast-FEL is able to identify many of the same key differences found by previous analyses, while often revealing additional sites and level of inferential detail.

## Results

### Establishing Method Performance

#### False Positives

**Fig. 1.**
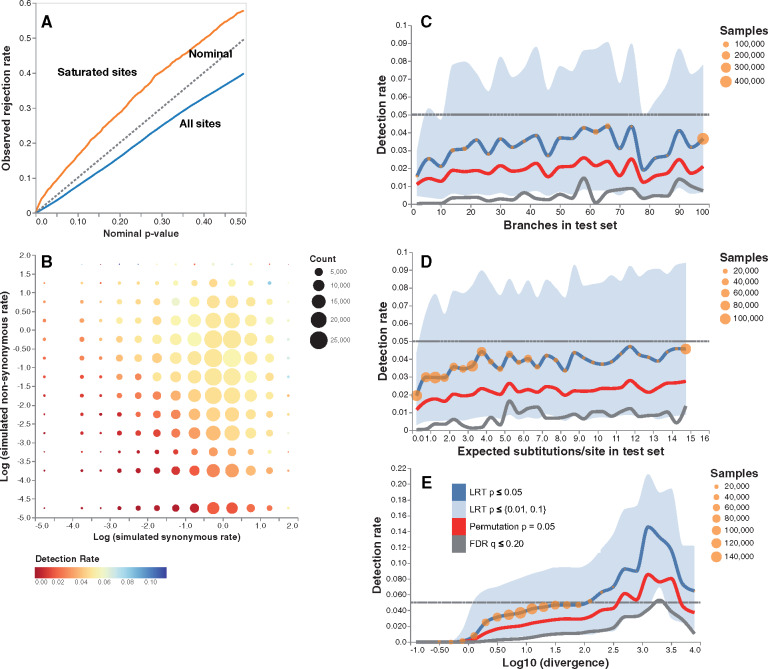
Contrast-FEL performance on null data (error control). The plots are based on 1,090,929 variable sites simulated with equal nonsynonymous rates on two branch sets (see text for simulation details). (*A*) Q–Q plots of LRT *P*-values for all sites (blue line) and for 3,684 *saturated* sites (${\;\text{log}\;}_{10}$ of the divergence level between 2.5 and 3.5, orange line). (*B*) Detection rate as a function simulated synonymous and nonsynonymous substitution rates (${\;\text{log}\;}_{10}$ scale). (*C*) Detection rate as a function of the number of branches in the test set (binned in increments of 5). Blue line: proportion of sites with LRT *P *<* *0.05, red line: proportion of sites with permutation *P *<* *0.05, gray line: proportion of sites with *q *<* *0.20. Blue area plot shows for the proportion of sites with LRT *P *<* *0.01 (lower) and LRT *P *<* *0.1 (upper). Orange circles reflect the number of sites contributing to each bin. (*D*) Detection rate as a function of the total branch length of the test set of branches (binned in increments of 0.5); same notation as in (*C*) otherwise. (*E*) Detection rate as a function of the ${\;\text{log}\;}_{10}$ of the divergence level at the site (binned in increments of 0.25); same notation as in (*C*) otherwise.

The rejection rate on null data with two branch sets, that is, on all sites where the evolutionary rates among branch sets were equal, in aggregate, was slightly lower than nominal rates (diagonal line, the test statistic performs exactly as expected under the null model; fig. 1*A*). We restricted the calculations only to variable sites because Contrast-FEL returns null results on invariant sites by definition (all maximum likelihood estimates for rate parameters are 0 at such sites) and because including invariant sites would only lower observed rejection rates. Contrast-FEL may become anticonservative (rejection rates above nominal) for very high divergence rates (fig. 1*A* and *E*, see below). The rate of false positives on null sites was largely independent of the values of synonymous and nonsynonymous rates, the levels of mean sequence divergence (within reason) in branch sets, and data set size (fig. 1*B*–*D*). The test is more conservative for low rates and smaller sets of branches, as expected. Permutation *P*-values and false discovery rate (FDR) *q*-values delivered more conservative detection rates than standard LRT *P*-values but mirrored the trends of the latter (fig. 1*C*–*D*). This is expected because permutation *P*-values are *only* computed conditioned on the significant LRT, so they can only be more conservative, and *q*-values incorporate a multiple testing correction. When a site is very saturated, that is, the product of the maximal rate estimate (*α* or *β*) and the alignment-wide tree length in expected substitutions per site exceeds 100, the test becomes anticonservative; the *q*–*q* plot for the sites with ${\;\text{log}\;}_{10}$ divergence rate between 2.5 and 3.5 is shown in figure 1*A* as those are the saturated sites with a detection rate permutation *P* ≥ 0.05 as per figure 1*E*. Our implementation reports the total branch length for each tested site, and saturated sites can be screened out using this metric. Such sites are rare in simulated data and should be even more rare in empirical alignments (we did not detect any in the empirical data).

Only 1 in 100 simulated data sets showed false positive rates (FPRs) of 8% or greater (10% or greater for 1 in 1,000), implying that one rarely encounters a simulated data set where the FPR is notably above the typical level.

#### Precision and Recall

The ability of Contrast-FEL to identify sites that experience differential selective pressures is influenced by the effective sample size, which depends in turn on the number of branches in the group and the extent of sequence divergence, and the effect size, that is, the magnitude of differences in nonsynonymous substitution rates, *β*. For simulations with two sets of branches, restricted to detectable sites (i.e., sites that were not invariable), power to detect differences aggregated over simulation scenarios is summarized in table 2. Over all detectable sites, the power using the FDR of 20% is 0.319. Restricted to the sites where the difference in *β* rates between groups was at least 1 (“Large effect”), the power rises to 0.603, and further restricting to only those sites where both the test and the background branch sets had at least three expected substitutions per site (“Large sample size”), increases the power to 0.860 see 3. Similar trends occur for testing using LRT *P*-values, or permutation-based *P*-values. Perfectly ladder-like trees on average yield somewhat higher power than either perfectly balanced or random/biological trees.

**Table 2.** Power of Contrast-FEL for Detecting Differences in Selection.

Simulation	*N*	p≤0.05	q≤0.2	**Permutation** p≤0.05
Overall	139,753	0.418	0.319	0.361
Large effect	30,923	0.727	0.603	0.665
Large effect/sample size	14,265	0.902	0.860	0.867
Perfectly balanced trees	39,671	0.411	0.316	0.354
Perfectly ladder-like trees	27,439	0.479	0.378	0.417
Random/biological trees	72,643	0.399	0.298	0.343
Four-class simulations				
Overall (omnibus)	18,141	0.355	0.276	0.379
Overall (any test)	18,141	0.415	N/A	N/A
Large effect (omnibus)	8,684	0.516	0.411	0.542
Large effect (any test)	8,684	0.587	N/A	N/A

Note.—*N*, total number of differentially selected sites in the set. Large effect is defined as having the absolute difference in simulated *β* rates of at least 1. Large sample size is defined as having at least three substitutions occurring along both test and reference branch sets.

**Fig. 2.**
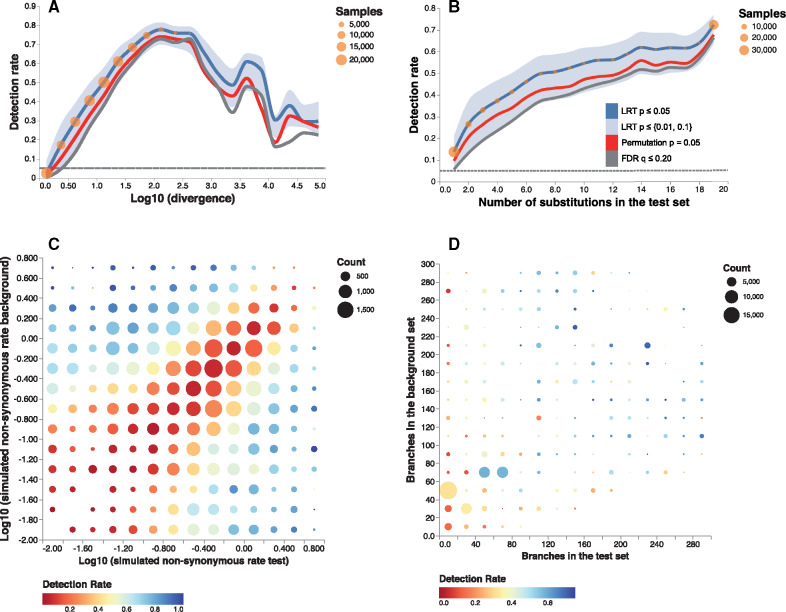
Contrast-FEL performance data with rate differences (power). The plots are based on 139,753 variable sites simulated with unequal nonsynonymous rates on two branch sets (see text for simulation details). (*A*) Detection rate as a function of the ${\;\text{log}\;}_{10}$ of the divergence level at the site. Blue line: proportion of sites with LRT *P *<* *0.05, red line: proportion of sites with permutation *P *<* *0.05, gray line: proportion of sites with *q *<* *0.20. Blue area plot shows for the proportion of sites with LRT *P *<* *0.01 (lower) and LRT *P *<* *0.1 (upper). Orange circles reflect the number of sites contributing to each bin. (*B*) Detection rate as a function of the number of inferred substitutions in the test set; same notation as in (*A*) otherwise. (*C*) Detection rate as a function of the simulated nonsynonymous rates in test and background branch sets and (*D*) the numbers of branches in the test and reference set.

The power of the Contrast-FEL adheres to the expected patterns; it increases with the sample size and the effect size. For example, greater levels of divergence at a site (up to a point) corresponded to notable gains in the power of the test (fig. 2*A*), as did greater numbers of substitutions occurring in the test set of branches, with power rising from ∼26.4% (p≤0.05) for two substitutions to 50.7% for eight substitutions (fig. 2*B*). Best power is achieved when the difference between substitution rates on the two sets of branches is large (fig. 2*C*), exceeding 80% for sufficiently disparate rates, and dropping to <10% for rates that are very similar. Power numbers are high when the size of either of the sets is not too small (fig. 2*D*).

**Fig. 3.**
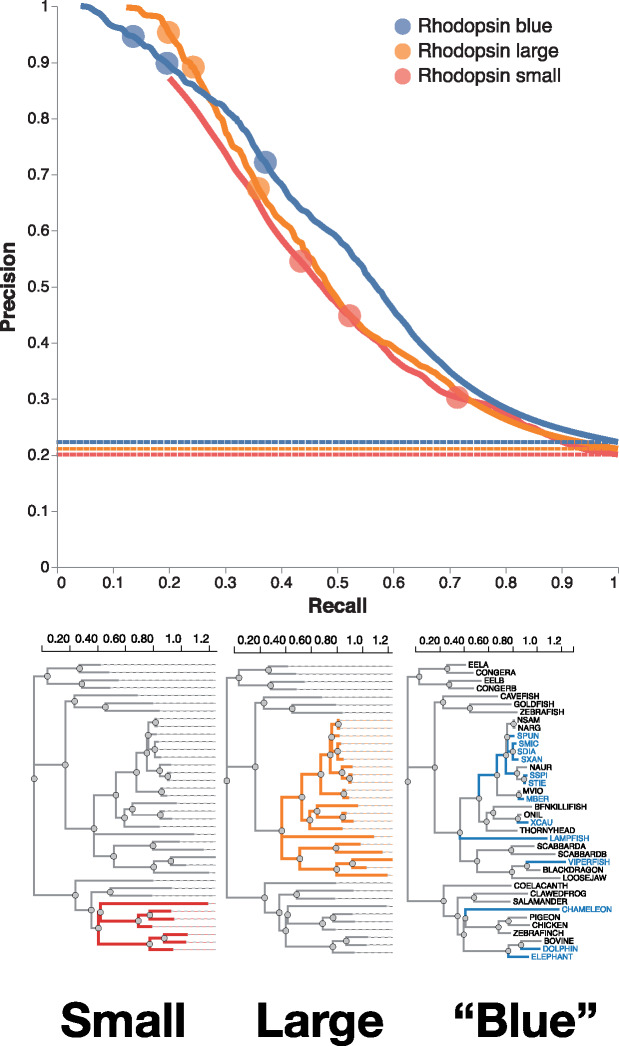
Contrast-FEL performance on vertebrate rhodopsin simulations. Precision–recall curves for the three sets of simulations, all based on the vertebrate rhodopsin tree from Yokoyama et al. (2008), with different choices for the “test” branch set (precision = true positives/all test positives, recall = true positives/positive training cases). Dotted lines show corresponding base rates for “no-skill” classifiers in each case (i.e., classify all sites as differentially selected). Circles on the individual curves show (left-to-right) precision–recall values for q=0.1,q=0.2,q=0.5. There were a total of 37,565 variable sites for the “small” case, 15,010 sites for the “large” case, and 37,401 sites for the “blue” case.

Next, we focus on the data simulated with the relatively small (31 sequences) biological tree of vertebrate rhodopsins from Yokoyama et al. (2008) and three different test branch sets: small clade, large clade, and branches grouped by phenotype (absorption wavelength), shown in figure 3. For sufficiently stringent FDR (*q*-values) cutoffs, high (90%) precision (positive predictive value [PPV]) can be achieved for all three cases, although the cutoffs need to be more stringent for the small clade scenario. High precision is achieved at the cost of fairly low recall (20−25%), and the small clade scenario has the worst performance among the three scenarios considered.

#### Four Branch Classes

**Fig. 4.**
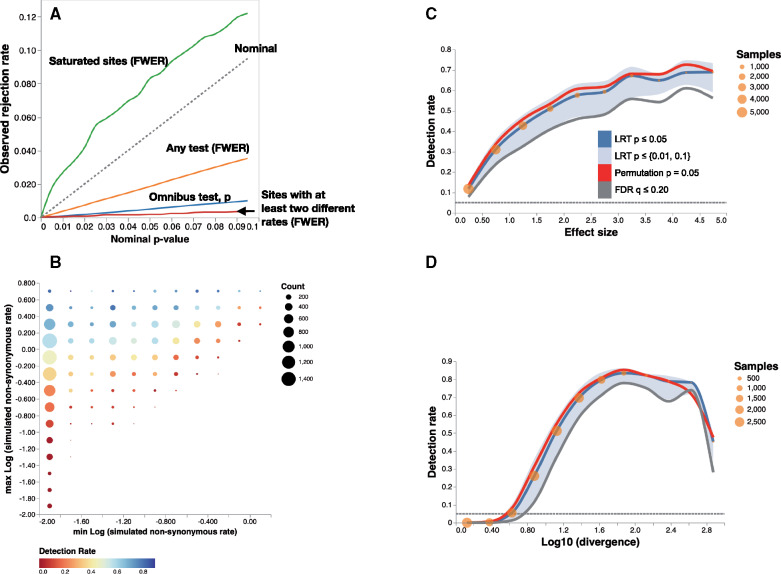
Contrast-FEL performance data with rate differences using four branch sets. (*A*) Q–Q plots of either omnibus test *P*-values (blue) or FWER (orange), which are based on rejections of any of the true nulls among 151,838 sites simulated where all branches had the same nonsynonymous rate. Green line shows the Q–Q plot of the FWERs on 1,944 *saturated* sites (${\;\text{log}\;}_{10}$ of the divergence level above 2.5). Red line shows the FWER for the 3702 data sets where some, but not of the nulls were true (i.e., some branch sets shared rates, whereas others did not). (*B*) Detection rate as a function of the ${\;\text{log}\;}_{10}$ of the lowest and highest nonsynonymous rates (rates lower than 0.01 are shown as 0.01) computed on 18,141 sites where at least two rates were different. (*C*) Detection rate as a function of the effect size, measured as the maximum difference between nonsynonymous rates among branch classes. Blue line: proportion of sites with LRT *P *<* *0.05 (the omnibus test), red line: proportion of sites with permutation *P *<* *0.05, gray line: proportion of sites with *q *<* *0.20. Blue area plot shows for the proportion of sites with LRT *P *<* *0.01 (lower) and LRT *P *<* *0.1 (upper). Orange circles reflect the number of sites contributing to each bin. (*D*) Detection rate as a function of the ${\;\text{log}\;}_{10}$ of the divergence level at the site.

Contrast-FEL remained conservative on null data when we applied it to alignments simulated with four branch classes (fig. 4*A*), for all types of tests: FWER (family-wise error rates) corrected pairwise differences, omnibus test (any rates are different), and when considering simulations where only some (but not all) of the groups had equal rates. As was the case with simpler two-class simulations, Type I error for severely saturated sites was somewhat elevated. Power to detect differences among any pair of branch groups, either via the pairwise or the omnibus test was strongly influenced by the effect size, ranging from near 0 for rates that were close in magnitude to over 80% for sites where the largest substitution rate was sufficiently high (>1), and sufficiently different (e.g., 5×) larger than the smallest rate (fig. 4*B*). Power of the method is strongly influenced by the effect size, that is, the magnitude of differences between *β* rates (fig. 4*C*), and the information content or saturation of the site, measured as a function of expected substitutions per site (fig. 4*D*). Introducing multiple branch classes increases the number of tests performed at each site, and because of the site-level multiple test correction, dilutes the power compared with the two-class case (table 2). Calling a site differentially evolving if any of the tests returns a significant corrected *P*-value realizes a 5–6% power boost compared with relying only on the omnibus test.

If a differentially evolving site was identified as such by the omnibus test at p≤0.05 (FWER corrected), in 99.6% of cases it was also identified by one or more of the individual pairwise tests, implying that in most cases (at least for our simulation), it is possible to pinpoint specific pairs of branch sets that are responsible. For the remaining 0.4% of sites, the omnibus test was significant, but none of the individual tests was. Alternatively, among the sites that were identified by at least one of the pairwise tests, 85.2% of them are also identified by the omnibus test; for the remainder of the sites, the omnibus test is not significant. Among those sites, 89.6% had a single pairwise significant test (median omnibus corrected *P *=* *0.103, interquartile range [0.072−0.159]), 9.5% had two pairwise significant tests (0.066[0.056−0.081]), and 0.9% had three pairwise significant tests (0.058[0.053−0.071]).

To boost power in low information settings (small branch sets or low divergence), it may be advisable to run only the omnibus test, that is, forego pairwise tests and the attendant FWER correction.

### Comparison with Post Hoc Tests

A reasonable heuristic approach for identifying sites that evolve differently between branch sets, *B*_1_ and *B*_2_ is to run an existing test which can determine whether the site evolved *nonneutrally* along either sets, and call the site differentially selected if there is evidence of positive selection on one group but not another. Approaches like this have been commonly used in literature, for example, Kapralov et al. (2012). One can also call a site differentially evolving if nonneutrality tests of *B*_1_ and *B*_2_ return *discordant results*. For example, *B*_1_ is negatively selected, but *B*_2_ is neutral, or *B*_2_ is positively selected and *B*_1_ is negatively selected. Contrast-FEL is, of course, a direct test of rate differences, so it could additionally identify, for instance, sites where *B*_1_ and *B*_2_ are both negatively/positively selected, but not at the same level. To illustrate the benefit of Contrast-FEL compared with the *Post hoc approach* (which also requires at least two separate computational analyses, one for each branch set, so may be less computationally efficient), we performed post hoc analyses based on independent FEL tests (one for each branch set) on a subset of 185,070 sites from 425 alignments.

Using the LRT *P*-value cutoff of 5%, over all variable sites, Contrast-FEL achieves FPR of 3.4%, power of 37.2%, PPV of 62.0%, and negative predictive value (NPV) of 91.1%. The “discordant results” post hoc FEL approach by comparison has FPR of 53.0%, power of 63.6%, PPV of 15.2%, and NPV of 89.6%; the dramatic increase in the rate of false positives for the post hoc method is mostly (93.7%) due to cases, where a site that was simulated under the null is misclassified because one of the branch sets is determined to be nonneutral by FEL and the other—neutral. All of the sites that were identified by Contrast-FEL but not by the post hoc heuristic were those where FEL (correctly) determined that both branch sets were conserved, but the degrees of conservation, measured by the $\beta_{i}/\alpha <1$ ratio, were different. Empirical data sets analyzed in the following section provide concrete examples of such sites which are labeled CC meaning they are conserved on the treatment set and conserved on the naive set (for more information, see table 3 or 4).

**Table 3.** Sites Evolving Differentially between the Treated and the Naive Sets in the HIV-1 RT Data Set, at p≤0.05.

		*β* (substitutions)				Standard FEL *P*-Value		
Codon	*α*	Treated	Naive	*P*-Value	*q*-Value	Treated	Naive	FEL Pattern
44	1.31	0.00 (9)	1.13 (8)	0.0286	0.799	0.003(−)	0.885(−)	CN
*65*	1.16	2.12 (11)	0.00 (3)	0.0156*	0.655	0.226(+)	0.075(−)	NN
*67*	1.24	1.39 (20)	0.00 (3)	0.0207*	0.694	0.792(+)	0.024(−)	NC
*70*	1.31	1.56 (17)	0.00 (5)	0.0374*	0.963	0.737(+)	0.051(−)	NN
*75*	0.86	1.80 (15)	0.00 (4)	0.0161*	0.600	0.130(+)	0.087(−)	NN
*100* ^a^	1.56	3.26 (29)	0.00 (13)	0.0150	0.836	0.094(+)	0.075(−)	NN
*103* ^a^	1.47	36.51 (104)	0.00 (7)	0.0000*	0.000	0.000(+)	0.073(−)	PN
*151* ^a^	0.93	2.67 (10)	0.00 (8)	0.0150*	0.719	0.023(+)	0.124(−)	PN
*181* ^a^	3.32	4.41 (21)	0.00 (7)	0.0010*	0.083	0.442(+)	0.004(−)	NC
*184* ^a^	0.00	8.29 (58)	0.34 (1)	0.0000*	0.000	0.023(+)	0.110(−)	PN
*188* ^a^	0.18	2.99 (14)	0.00 (0)	0.0061	0.411	0.000(+)	0.491(−)	PN
*190* ^a^	1.52	3.41 (33)	0.00 (10)	0.0004*	0.041	0.031(+)	0.011(−)	PC
*215* ^a^	0.44	1.50 (12)	0.00 (13)	0.0255*	0.775	0.021(+)	0.199(−)	PN
228^a^	1.53	1.30 (21)	0.00 (9)	0.0436	0.974	0.753(−)	0.019(−)	NC
302	0.62	0.00 (1)	8.05 (3)	0.0420*	1.000	0.458(−)	0.054(+)	NN

Note.—*α*, maximum likelihood estimate (MLE) of the site-specific synonymous rate; *β*, nonsynonymous rate.

* Permutation *P*-value is ≤0.05; substitutions are counted along branches in the corresponding set using joint maximum likelihood inference of ancestral states under the site-level alternative model, codons in italics are known to be involved in drug resistance (Rhee et al. 2003).

a Codon identified as directionally evolving in table 2 of Murrell et al. (2012). FEL *P*-values are computed by separately testing for nonneutral evolution on the corresponding set of branches, with the + or - sign indicating the nature of selection (positive or negative). FEL pattern encodes the inferred pattern of evolution for treated/naive branches: P, positively selected (at p≤0.05); C, conserved; N, neutral; for example, PC means “positively selected” on the treated set, and “conserved” on the naive set.

The heuristic in which sites are called differentially evolving where only one of the sets was under positive selection (the method commonly used in literature) has FPR of 3.3%, much reduced power of 17.1%, NPV of 94.6%, and PPV of 25.9%.

### Exploring the Effect of Model Misspecification

To see whether the performance of Contrast-FEL suffers when data are generated under models that make different parametric distributions, we fitted Contrast-FEL to data generated under a branch-site model (Wisotsky et al. 2020), where *ω* rates vary from site to site and branch to branch, but using a random effect, that is, set of branches fixed a priori are not expected to show detectably different patterns of site-level *ω*. This model allows independent unrestricted rate variation between branches and sites and is computationally faster and less parameter-rich than covarion-type models that allow complex correlation structures between rates (see discussion in Murrell et al. [2015]). Using the simulation scenario (CV3o6, see http://data.hyphy.org/web/busteds/), and the branch partition shown in supplementary figure S1, Supplementary Material online, we obtained a slightly elevated rate of 0.07 false positives with LRT at nominal *P *=* *0.05, suggesting that the LRT is misattributing some “random” rate variation to fixed branch partitions. However, the permutation *P*-value test, which we designed as a nonparametric guard to correct for some model misspecification, maintains a nominal rate of 0.045 for *P *=* *0.05 (see supplementary fig. S2, Supplementary Material online). Similarly, no elevated false positives are seen with multiple-test corrected *q*-values. Because of the limited scope of these simulations, we cannot draw general conclusions about robustness to model misspecification.

### Empirical Data

#### DR in HIV-1 Reverse Transcriptase

We applied Contrast-FEL to an alignment of 476 HIV-1 reverse transcriptase (RT) sequences with 335 codons isolated from 288 HIV-1 infected individuals, previously analyzed in Murrell et al. (2012). There were two sequences sampled from each individual: one prior to treatment with RT inhibitors and one following treatment. We partitioned the branches in the tree into three groups: pretreatment terminal branches (*naive*), posttreatment terminal branches (*treated*), and the rest of the branches (nuisance group, supplementary fig. S1, Supplementary Material online, HIV-1 RT). Because we expect that the primary difference between the selective regimes on *naive* versus *treated* branches is due to the action of the antiretroviral drugs, most of the sites that have detectable differences in selective pressures should be implicated in conferring DR. Using nominal *P*-value cutoff of 0.05, Contrast-FEL identifies 15 sites that evolve differentially, between which 12 are known DR sites, achieving PPV of 0.8. Of the three non-DR sites that are found, codons 44 and 302 are actually more conserved (lower *β*) in the treated group, which is a different mode of selective pressure than positive selection exerted by antiretroviral drugs. They are also not supported by the permutation test, which could indicate that these sites are picked up due to sampling variation. Among the 12 DR sites identified by LRT *P*-values, ten are also supported by the permutation test—an indicator of robustness to branch sampling artifacts. The most conservative approach, based on FDR corrected *q*-values of 0.20 or lower, identifies four codons: 103, 181, 184, and 190, all of which are on the list of canonical escape mutations with very strong phenotypic effects (Rhee et al. 2003). All of these sites have many inferred mutations in the treated group, and large differences between inferred *β* rates, which places them in the large effect/large sample size category. As a comparison with one common practice to screen for differential selection today, we also used fixed effects likelihood (FEL; Kosakovsky Pond and Frost 2005) to test each branch set for evidence of deviations from neutrality (either positive or negative selection). For site 190, subject to differential selection with a large effect size, FEL reveals that the treated branches experience positive selection (FEL p≤0.05), and naive branches—negative selection. However, no other sites show such a clean pattern. For sites 103, 151, 184, and 215, test branches are subject to positive selection (FEL p≤0.05), whereas naive branches evolve neutrally. For sites 67, 181, and 228, naive branches are subject to negative selection (FEL p≤0.05), whereas test branches evolve neutrally. For the remainder of the sites, neither branch class evolves in a way that is detectably different from neutrality according to FEL. This comparison highlights that comparing the results of two independent tests applied to subsets of the data to detect evolutionary differences is statistically suboptimal.

The performance of Contrast-FEL (a generalist method) in identifying sites of phenotypic relevance compares favorably with the performance of a purpose-built Model of Episodic Directional Selection (MEDS) method (Murrell et al. 2012), designed to find directional evolution along selected branches. MEDS identified 17 sites of which 10 were known DR sites (PPV of 58.9%, see table 2 in Murrell et al. (2012), and both methods agreed on nine sites. Of course, unlike MEDS, Contrast-FEL is not able to identify specific residues that may be the targets of selection at specific sites.

#### Selection on HIV-1 Envelope Conditioned on the Route of Transmission

We reanalyzed an alignment of 131 partial HIV-1 envelope (no variable loops) sequences with 806 codons from Tully et al. (2016); these sequences were isolated from acute/early infections and represent “founder” viruses. These sequences were labeled by whether or not the infected individual was infected via a heterosexual (HSX) exposure, or men who have sex with men (MSM) exposure; interior branches were labeled as HSX or MSM if all of their descendants had the same label, and were left unlabeled (nuisance set) otherwise. Tully et al. (2016) found gene-wide differences in selection among branch groups (a larger proportion of sites, but subject to weaker positive selection, on HSX branches compared with MSM branches), using the RELAX test (Wertheim et al. 2015), but lacked the framework to pinpoint specific residues that evolved differentially. Contrast-FEL identified 32 differentially selected sites (*P*-value) of which three passed the FDR correction (table 4). One of these sites, 626, is conserved in both branch sets, but at a stronger level (lower *β*) in the MSM set, whereas another (786) is positively selected in both, but at a stronger level on HSX branches.

**Table 4.** Sites Evolving Differentially between the HSX and the MSM Sets in the HIV-1 Env Data Set from Tully et al. (2016).

Codon	*α*	*β* (substitutions)HSX	MSM	*P*-Value	*q*-Value	Standard FEL *P*-ValueHSX	MSM	FEL Pattern
49	0.56	0.12 (2)	0.69 (12)	0.0365*	1.000	0.151(−)	0.742(+)	NN
50	0.30	0.61 (6)	0.00 (2)	0.0009*	0.177	0.331(+)	0.015(−)	NC
53	0.88	0.00 (2)	0.32 (9)	0.0391	1.000	0.002(−)	0.088(−)	CN
142	4.13	5.09 (34)	2.86 (37)	0.0497	1.000	0.373(+)	0.338(−)	NN
158	0.62	2.24 (9)	0.84 (10)	0.0168	0.905	0.010(+)	0.582(+)	PN
197	1.88	2.93 (20)	1.17 (23)	0.0053*	0.616	0.361(+)	0.287(−)	NN
233	0.20	0.25 (3)	0.00 (1)	0.0337*	1.000	0.836(+)	0.046(−)	NC
264	0.33	1.91 (11)	0.65 (9)	0.0107	0.859	0.005(+)	0.427(+)	PN
275	2.45	4.16 (28)	1.71 (28)	0.0031	0.498	0.128(+)	0.344(−)	NN
303	1.63	6.98 (34)	3.55 (39)	0.0093*	0.837	0.000(+)	0.082(+)	PN
336	3.80	1.09 (11)	2.47 (35)	0.0254	1.000	0.009(−)	0.297(−)	CN
344	0.40	1.25 (8)	0.31 (7)	0.0074*	0.742	0.042(+)	0.682(−)	PN
408	0.52	0.70 (4)	1.80 (22)	0.0436	1.000	0.663(+)	0.007(+)	NP
442	0.00	0.31 (1)	0.00 (0)	0.0362*	1.000	0.031(+)	1.000(−)	PN
530	0.69	0.29 (2)	0.00 (3)	0.0359*	1.000	0.305(−)	0.002(−)	NC
572	1.55	4.01 (29)	2.27 (33)	0.0469	1.000	0.046(+)	0.456(+)	PN
574	1.10	0.52 (6)	0.10 (5)	0.0389	1.000	0.230(−)	0.002(−)	NC
598	0.59	1.13 (9)	0.28 (7)	0.0410	1.000	0.235(+)	0.255(−)	NN
626	12.77	4.14 (37)	1.40 (41)	0.0003*	0.120	0.001(−)	0.000(−)	CC
672	2.46	3.15 (25)	1.46 (37)	0.0139*	0.799	0.433(+)	0.079(−)	NN
683	1.88	2.05 (15)	0.85 (17)	0.0404*	1.000	0.786(+)	0.039(−)	NC
685	0.00	1.16 (9)	0.12 (2)	0.0008*	0.213	0.001(+)	0.259(+)	PN
690	1.24	0.28 (5)	1.33 (18)	0.0176*	0.837	0.050(−)	0.983(+)	CN
702	0.00	1.13 (8)	0.28 (4)	0.0174*	0.877	0.002(+)	0.101(+)	PN
703	1.31	1.27 (7)	0.13 (11)	0.0119*	0.739	0.958(−)	0.003(−)	NC
720	0.49	1.47 (11)	0.44 (13)	0.0107	0.785	0.026(+)	0.860(−)	PN
722	0.00	0.13 (1)	0.78 (9)	0.0325	1.000	0.288(+)	0.007(+)	NP
734	0.50	2.23 (14)	0.75 (10)	0.0111	0.749	0.005(+)	0.536(+)	PN
773	0.25	0.00 (0)	0.31 (7)	0.0406	1.000	0.093(−)	0.778(+)	NN
781	0.51	0.50 (5)	0.00 (2)	0.0031*	0.416	0.975(−)	0.005(−)	NC
786	1.40	6.67 (24)	3.33 (38)	0.0274	1.000	0.000(+)	0.009(+)	PP
804	0.25	0.00 (0)	1.53 (15)	0.0000*	0.031	0.119(−)	0.002(+)	NP

Note.—Other notation the same as in table 3.

#### Cell Shape in Epidermal Leaf Trichomes

Mazie and Baum (2016) investigated which codons in a developmentally important gene (BRT) in Brassicaceae (58 sequences, 318 codons) may be associated with the evolution of a different trichome cell shape in the genus *Physaria*. Using gene-level mean differences in *ω* between subsets of branches, they identified that the average strength of selection is different in *Physaria* compared with the rest of the taxa. They then used a revised restricted branch-site (Zhang et al. 2005) method to detect ten codons that were subject to positive selection in the *Physaria* clade and four codons were “distinctive” (majority amino acid was different in *Physaria*), but not positively selected. Contrast-FEL identified 29 differentially selected codons at p≤0.05 (18 at q≤.20), including all ten positive codons from Mazie and Baum (2016) and one out of four “distinctive” codons (table 5). Given the general conservative nature of Contrast-FEL, it is reasonable to assume that it is more powerful (rather than prone to making more Type I errors) than the original test which was limited to a  50% subset of branches and used much more stringent parametric assumptions on rate distributions, including shared negative selection regimes on background branches, and a single *ω* to account for the positive selection rate class.

**Table 5.** Sites Evolving Differentially between *Physaria* and Other Taxa in the BLT Gene from Mazie and Baum (2016).

Codon	*α*	*β* (substitutions)*Physaria*	Other	*P*-Value	*q*-Value	Standard FEL *P*-Value*Physaria*	Other	FEL Pattern
4	0.00	80.00 (2)	0.76	0.0320	0.392	0.018(+)	0.220(+)	PN
43	0.69	4.27 (1)	0.00	0.0110*	0.194	0.251(+)	0.043(−)	NC
60	0.00	57.88 (1)	0.82	0.0265	0.421	0.020(+)	0.140(+)	PN
107	0.93	1.70 (3)	0.16	0.0123*	0.206	0.511(+)	0.108(−)	NN
122	0.21	0.00 (0)	1.40	0.0308*	0.426	0.476(−)	0.094(+)	NN
126	1.62	0.89 (2)	0.08	0.0467	0.550	0.563(−)	0.017(−)	NC
153^a^	0.24	4.17 (6)	0.23	0.0001*	0.006	0.001(+)	0.960(−)	PN
156	0.66	2.91 (7)	0.67	0.0283*	0.409	0.051(+)	0.986(+)	NN
163	0.35	1.31 (2)	0.12	0.0473	0.538	0.276(+)	0.476(−)	NN
164	0.32	2.04 (3)	0.25	0.0267	0.404	0.088(+)	0.847(−)	NN
167^a^	0.37	6.54 (10)	0.70	0.0000*	0.003	0.001(+)	0.549(+)	PN
169	5.04	0.74 (4)	0.07	0.0476	0.522	0.016(−)	0.000(−)	CC
171^a^	2.03	4.00 (4)	0.00	0.0000*	0.003	0.320(+)	0.000(−)	NC
173^a^	0.47	4.12 (6)	0.36	0.0011*	0.044	0.026(+)	0.820(−)	PN
174^a^	0.00	11.60 (8)	0.31	0.0000*	0.000	0.000(+)	0.346(+)	PN
175^a^	0.85	3.67 (7)	0.00	0.0000*	0.002	0.046(+)	0.006(−)	PC
176	0.56	1.22 (1)	0.00	0.0057*	0.130	0.450(+)	0.025(−)	NC
178	1.29	1.93 (3)	0.12	0.0085	0.160	0.636(+)	0.026(−)	NC
179	0.77	1.85 (2)	0.00	0.0008*	0.038	0.306(+)	0.008(−)	NC
180^a^	0.17	2.22 (3)	0.00	0.0001*	0.006	0.008(+)	0.161(−)	PN
187	0.46	0.91 (2)	0.00	0.0066*	0.141	0.503(+)	0.023(−)	NC
188	1.31	1.03 (3)	0.00	0.0075*	0.149	0.790(−)	0.001(−)	NC
190^a^	0.00	1.47 (4)	0.07	0.0015*	0.053	0.020(+)	0.562(+)	PN
191^a^	0.45	2.49 (4)	0.13	0.0025*	0.080	0.046(+)	0.302(−)	PN
198	0.66	2.17 (2)	0.00	0.0026*	0.076	0.210(+)	0.023(−)	NC
255^a^	2.06	1.47 (3)	0.07	0.0047*	0.124	0.636(−)	0.000(−)	NC
262	1.43	1.27 (3)	0.17	0.0320*	0.407	0.872(−)	0.009(−)	NC
270	2.32	0.87 (3)	0.00	0.0047*	0.115	0.248(−)	0.000(−)	NC
278^b^	1.64	0.86 (1)	0.00	0.0320*	0.424	0.567(−)	0.001(−)	NC

Note.—Other notation the same as in table 3.

a Codon identified as positively selected in *Physaria* (table 3 of Mazie and Baum [2016]).

b Codon identified as “distinctive” in *Physaria* (table 4 of Mazie and Baum [2016]).

#### Evolution of Rubisco in C3 versus C4 Photosynthetic Pathway Plants

Several studies comparing evolutionary selective pressures on the *rbcL* gene in C3 and C4 plants have identified several sites that appear to be under positive selection in either C3 or C4 plants (Kapralov and Filatov 2007; Kapralov et al. 2012), as well as several others that have different targets for directional evolution based on the pathway (Parto and Lartillot 2018). In this alignment of 179 sequences and 447 codons, Contrast-FEL identified 15 sites that evolve differentially between C3 and C4 plants (LRT p≤0.05), of which six had been previously identified as being subject to differential directional selection by a mutation-selection model, and five additional sites were identified by this model (cf. table 6). An interesting example in this data set is site 309 which was found as positively selected previously, but is classified as conserved in both C3 and C4 plants by FEL; this appears to be a result of the high synonymous rate inferred at the site, which is a hallmark false positive for standard selection analyses that ignore site-to-site synonymous rate variation (Kosakovsky Pond and Muse 2005; Wisotsky et al. 2020). However, a weaker extent of conservation in C4 plants is inferred by Contrast-FEL at this site.

**Table 6.** Sites Evolving Differentially between C3 and Other Taxa in the *r*bcL Gene.

Codon	*α*	*β* (substitutions)C3	C4	*P*-Value	*q*-Value	Standard FEL *P*-ValueC3	C4	FEL Pattern
23	0.00	0.87 (4)	0.00 (0)	0.0494	1.000	0.083(+)	1.000(−)	NN
86^a^	1.02	1.22 (7)	0.00 (1)	0.0164*	1.000	0.794(+)	0.039(−)	NC
249	0.98	0.99 (6)	0.00 (0)	0.0375*	1.000	0.995(+)	0.062(−)	NN
251	0.00	0.95 (5)	0.00 (0)	0.0169	1.000	0.342(+)	1.000(−)	NN
262^a^^,b^	0.48	4.15 (15)	1.15 (3)	0.0292*	1.000	0.005(+)	0.460(+)	PN
281^a^^,c^	0.00	0.44 (2)	3.90 (10)	0.0008*	0.372	0.216(+)	0.001(+)	NP
295	2.46	0.00 (1)	0.62 (4)	0.0482*	1.000	0.000(−)	0.084(−)	CN
309^a^^,c^	76.21	0.00 (0)	0.95 (5)	0.0047*	0.700	0.000(−)	0.013(−)	CC
315	1.43	0.00 (2)	0.72 (3)	0.0479*	1.000	0.008(−)	0.452(−)	CN
332	0.00	0.00 (1)	0.85 (2)	0.0472*	1.000	1.000(−)	0.101(+)	NN
354^a^	0.49	1.32 (6)	0.00 (1)	0.0160*	1.000	0.309(+)	0.187(−)	NN
367	0.96	1.21 (6)	0.00 (1)	0.0285	1.000	0.780(+)	0.074(−)	NN
439^a^^,b^	1.04	2.26 (10)	0.31 (4)	0.0143*	1.000	0.136(+)	0.216(−)	NN
443^a^^,b^	0.00	2.29 (9)	0.00 (0)	0.0018*	0.412	0.005(+)	1.000(−)	PN
456	3.15	0.00 (0)	0.72 (8)	0.0481*	1.000	0.000(−)	0.051(−)	CN

Note.—Other notation the same as in table 3.

a Codon reported as differentially evolving by mutation-selection directional DS3 model in Parto and Lartillot (2018).

b Codon identified as positively selected in C3 plants (^c^C4 plants) previously (table 1 of Parto and Lartillot [2018]).

#### Selection on Cytochrome B of Haemosporidians Infecting Different Hosts

Pacheco et al. (2018) performed an in-depth evolutionary analysis of three mitochondrial genes from 102 Haemosporidian parasite species partitioned into four groups based on the hosts. The analysis concluded that the genes were subject to mostly purifying selection, with different gene-level strengths of selection established using RELAX. For example, in the cytochrome B gene (376 codons) which we reanalyze here, selection in the plasmodium infecting avian hosts clade was intensified relative to the plasmodium infecting primate/rodent hosts. Because this analysis contained more than two branch groups, Contrast-FEL conducted seven tests per site—the omnibus test and six pairwise group comparisons (table 7). Overall, 28 sites showed evidence of differential selection with at least one test (FWER corrected), and five tests passed FDR correction for the omnibus test. For clarity, we did not consider FEL analyses on individual branch sets and only focused on Contrast-FEL inference. Twenty-two of 28 sites were detected by the omnibus and between one and three pairwise tests, whereas six sites were reported only by one of the pairwise tests, highlighting the additional resolution offered by these more focused tests. Patterns of differences at individual sites varied widely, with every possible pair being significantly different at least once. The simplest case (e.g., 160 and 179) is a significant discordance between two groups of branches. Another repeated pattern is when one group of branches stands apart from all others (e.g., 89 and 102).

**Table 7.** Sites Evolving Differentially among the Four Branch Groups in the Cytochrome B Mitochondrial Gene of Haemosporidians from Pacheco et al. (2018), According to the Omnibus Test or At Least One Pairwise Test at LRT Corrected *P*-value of ≤0.05.

Codon	*α*	*β* (substitutions) Haemoproteidae	Avian Hosts	Mammalian Hosts	Leucocytozoon	Other	*P*-Value	*q*-Value	Significant Pairwise Tests
56	3.81	0.00 (2)	0.00 (2)	0.00 (3)	0.75 (4)	0.00	0.0465*	0.795	AL, ML
89	0.94	0.00 (1)	0.00 (3)	0.00 (4)	1.33 (5)	0.00	0.0001*	0.017	HL, AL, ML
102	1.66	0.00 (2)	0.00 (2)	0.23 (5)	1.83 (6)	0.42	0.0014*	0.103	HL, AL, ML
150	0.79	0.00 (1)	0.00 (2)	1.27 (9)	0.00 (2)	0.56	0.0075*	0.403	AM, ML
158	0.42	0.00 (0)	0.00 (0)	0.00 (2)	1.30 (5)	0.00	0.0207*	0.518	AL, ML
160	1.25	0.94 (6)	0.00 (1)	0.08 (8)	0.43 (2)	0.75	0.0422	0.755	HA
179	0.89	0.48 (2)	0.00 (0)	0.25 (6)	1.46 (6)	1.10	0.0171*	0.495	AL
182	1.43	0.00 (0)	0.00 (0)	0.00 (0)	2.23 (9)	0.42	0.0001*	0.013	HL, AL, ML
183	1.80	0.00 (0)	0.00 (3)	0.08 (2)	1.20 (4)	0.54	0.0058*	0.361	HL, AL, ML
186	0.00	0.22 (1)	0.00 (0)	0.17 (2)	0.65 (3)	0.16	0.3127*	1.000	AL
193	1.53	0.00 (0)	0.00 (5)	0.00 (1)	1.07 (5)	0.00	0.0147*	0.462	AL, ML
194	1.05	0.00 (0)	0.00 (0)	0.00 (1)	0.49 (4)	0.19	0.1347*	1.000	ML
222	0.64	0.00 (0)	0.00 (0)	0.00 (0)	0.70 (4)	0.00	0.0246*	0.514	AL, ML
223	1.45	0.48 (3)	0.00 (0)	0.00 (1)	0.70 (6)	0.00	0.0200*	0.538	AL, ML
248	0.48	0.00 (0)	0.10 (1)	1.01 (9)	0.22 (1)	0.64	0.0573*	0.937	AM
253	1.67	0.00 (4)	0.00 (4)	0.00 (6)	0.95 (4)	0.40	0.0132*	0.452	AL, ML
256	1.92	0.00 (6)	0.00 (3)	0.73 (8)	0.00 (0)	0.64	0.0386*	0.725	AM
283	0.38	0.00 (1)	0.16 (1)	0.00 (0)	1.36 (6)	0.64	0.0241*	0.534	ML
285	1.17	0.22 (1)	0.00 (1)	0.39 (5)	1.44 (6)	1.01	0.0852*	1.000	AL
289	3.28	0.00 (0)	0.00 (1)	0.00 (0)	1.31 (3)	0.00	0.0012*	0.110	HL, AL, ML
309	1.05	0.83 (2)	0.36 (6)	1.11 (9)	4.24 (8)	0.61	0.0822*	1.000	AL
310	1.96	0.22 (1)	0.00 (1)	0.00 (2)	0.83 (4)	0.00	0.0119*	0.499	AL, ML
331	0.15	0.00 (0)	0.00 (0)	0.00 (0)	7.31 (9)	0.00	0.0000*	0.012	HL, AL, ML
338	0.00	0.00 (0)	0.00 (0)	0.00 (0)	0.98 (3)	0.00	0.0115	0.541	AL, ML
341	0.43	0.49 (3)	0.28 (2)	0.00 (2)	0.83 (6)	0.45	0.1118*	1.000	ML
343	0.25	0.97 (3)	0.14 (1)	0.00 (1)	1.23 (4)	1.05	0.0224*	0.527	HM, ML
351	1.75	0.00 (2)	0.00 (3)	0.91 (8)	0.88 (4)	0.25	0.0367*	0.727	AM
366	0.52	1.43 (5)	0.20 (3)	0.00 (1)	0.61 (6)	0.67	0.0123*	0.463	HM, ML

Note.—Individual pair tests in the last column and codes as follows. HA, Haemoproteidae versus Avian Hosts (one site); HM, Haemoproteidae versus Mammalian Hosts (two sites); HL, Haemoproteidae versus Leucocytozoon (six sites) AM, Avian Hosts versus Mammalian Hosts (four sites); AL, Avian Hosts versus Leucocytozoon (eighteen sites); ML, Mammalian Hosts versus Leucocytozoon (twenty sites). Other notation the same as in table 3.

## Discussion

To narrow down the genetic basis of adaptation, many studies contrast evolution between different subsets of branches in a phylogenetic tree, selected to represent different phenotypes, environments, or fitness regimes. As we sequence more organisms and obtain better annotations of function and phenotypic differences, such contrast analyses are likely to become more common. However, with a few exceptions, methods that researchers have adopted to find differentially evolving sites were not designed to explicitly test for such differences. The Contrast-FEL approach, presented here, addressed this methodological shortcoming, and establishes a formal statistical framework for testing for differences in evolutionary rates among two or more sets of branches at individual sites. Unlike approaches which infer something about each branch set separately (e.g., is branch set X under positive selection at site S?) and compare these inferences in a post hoc fashion (site S is selected on branch set X but not on set Y), Contrast-FEL enables direct rigorous testing for differences in nonsynonymous evolutionary rates at individual alignment sites in predefined nonoverlapping sets of branches and is computationally feasible for all but the largest comparative analyses. Contrast-FEL has good operating characteristics on data simulated under a broad range of scenarios and finds large numbers of differentially evolving sites in empirical data sets. When prior results are available, we find that in addition to recovering many sites with known effect on phenotype (e.g., HIV-1 DR), or sites found with alternative methods (*rbcL*, BRT), Contrast-FEL reports subtler differences, such as sites that are subject to differing degrees of conservation, or not subject to detectable positive or negative selection in either subset. Therefore, Contrast-FEL may enable more precise and powerful comparative analyses, and it is also the first method of this class that is able to compare selective pressures among more than two groups of branches. The general framework of site-level rate comparison using LRTs presented here can be readily extended to compare other types of evolutionary parameters, for example, rates that are informed by amino acid properties (Delport et al. 2010), rates of instantaneous substitutions that involve multiple nucleotides (Venkat et al. 2018), or rates that influence positional synonymous substitutions (Rubinstein et al. 2011)

As with any statistical inference method, it is important to appreciate when it will work well and when it will not. Since Contrast-FEL tests for significant differences in *ω* rates, a positive result means that the sites are subject to different selection intensities, for example, purifying versus neutral or positive diversifying, and not that they evolve toward different target residues (directional selection). It will work best on sufficiently large alignments with multiple substitutions in each of the branch sets, whereas, on small or genetically similar data sets, power will be low for all but the most dramatic differences. Our simulations provide guidelines for performance, and, if desired, power simulations for a specific data set can be used to determine what effect size can be realistically detected. As it is reasonable to assume that in most alignments most sites do not evolve differentially (or at least not dramatically so) along subsets of branches, care must be taken to control for false discovery. Our empirical data analyses on alignments with prior site-level results do not indicate a dramatically inflated rate of false positives (the lists of sites overlap substantially with those identified previously). On the other hand, being too conservative will lead to loss of power, because for site-level inference it does not grow with the length of the alignment (Scheffler et al. 2014).

Our simulations can be used as guideline for what power can be expected from a specific data set based on its size (number of branches, size of partitions) and information content (number of substitutions, divergence). For smaller data sets, only sites with strong effects (large differences in rates) are likely to be found (fig. 3). It is worth noting that by default Contrast-FEL will always perform the omnibus and report FWER corrected *P*-values, permutation *P*-values, and FDR corrected *q*-values. The main choice a user faces in terms of what tests are performed is if permutation *P*-values are computed. Depending on the desired tradeoffs between precision and recall, the user may choose to use site-level *P*-values (highest power, lowest precision), permutation *P*-values (intermediate power and precision, some degree of robustness to model misspecification), or *q*-values (lowest recall, highest precision).

Statistical models of evolution, including those presented here, are gross approximations of highly complex biological reality. Thus, they are likely to be misspecified for biological data. A positive/negative Contrast-FEL result should not be interpreted as dispositive evidence of the effect of branch partition (phenotypes, environments, etc.) affects selective regimes. It should instead be viewed as a hypothesis generation tool: Detected sites may present an attractive target for subsequent characterization using experiments or other approaches that do not solely rely on comparative data analysis (MacCallum and Hill 2006). Our simulations are extensive, but they only establish that the tests perform as expected when the data are generated under the assumed models in a broad range of settings (a necessary step). There are numerous violations which might negatively influence the test: background heterotachy (Jones et al. 2020), multinucleotide substitutions (Venkat et al. 2018), or the general tendency of some models to “absorb” unmodeled variation into estimable parameters (Jones et al. 2018). How branches are partitioned into groups will differ based on the nature of the question being asked: Sometimes it could be obvious (treated/untreated terminal branches) and sometimes highly ambiguous (discrete or discretized phenotype whose values of not observed for ancestral lineages). Misspecification of branch sets could lead to degraded test performance, although one approach offered by Contrast-FEL is to assign branches with uncertain labeling to the “ignore” group. Sensitivity to model violations is a general statistical inference issue, and establishing some degree of robustness in complex settings like phylogenetic codon models where analytical results are not available is usually accomplished by simulations, which are often limited in scope due to the computational expense and the imagination of the individuals designing model violations. We considered one possible misspecification due to heterotachy and found that the LRT based test was slightly anticonservative, but the permutation *P*-value test restored nominal behavior, suggesting that permutation *P*-values may be less sensitive to some model violations, although they do incur a noticeable additional computational expense.

## Materials and Methods

### Statistical Models and Parameter Inference

Our model adapts the FEL method (Kosakovsky Pond and Frost 2005), which has previously been used to find sites that evolve under different selective pressures in different alignments of the same gene (Kosakovsky Pond et al. 2006). Consider an alignment of *N* coding nucleotide sequences with *S* codons, and a given tree topology T with B≥N branches. Branches in the tree are partitioned into disjoint nonempty sets, $B_{i},\;i=1,2,\ldots ,K\geq 2$, and this partition is fixed a priori. If *K *>* *2, one of the branch groups can be designated as background and not explicitly tested.

Sequence evolution is modeled using the standard class of Muse–Gaut class of codon models (Muse and Gaut 1994), MG-REV-CF3×4. The rate matrix used to model codon sequence evolution follows the standard codon-substitution model structure:

$$q_{ij}(\alpha ,\beta ,\theta ,\Pi )=\{\begin{array}{cc} \alpha \theta_{ij}\pi_{j}, & \delta (i,j)=1,\;AA(i)=AA(j),\\ \beta_{i}\theta_{ij}\pi_{j}, & \delta (i,j)=1,\;AA(i)\neq AA(j),\\ 0, & \delta (i,j)>1,\\ -\sum_{l\neq i}q_{il}, & i=j. \end{array}$$

Here, δ(i,j) counts the number of nucleotide differences between codons *i* and *j*; *AA*(*x*) is the amino acid encoded by codon *x*; *θ* represents the underlying nucleotide substitution rate parameters (assumed to follow the general time-reversible form); Π are the equilibrium codon frequencies, obtained using the CF3 × 4 corrected empirical estimator (Kosakovsky Pond et al. 2010) with nine parameters; developed to correct estimation biases that arise when nucleotide frequencies are used to estimate codon frequencies without correcting for the absence of stop codons.

The key parameters of the model are: *α*—the site-specific synonymous substitution rate, and *β_i_*—the site-specific nonsynonymous substitution rate for branch group *i*, and all others are nuisance parameters (table 8). We fit the model to a coding sequence alignment using the following procedure.

Step 1: Obtain initial nuisance parameter estimates: infer B branch length parameters *t_b_*, and five nucleotide substitution rates $\theta_{AC},\theta_{AT},\theta_{CG},\theta_{CT},\theta_{GT}$ using the general time reversible nucleotide model.

Step 2: Infer initial codon tree scaling and group-level ω: fit the MG-REV-CF3×4 model to the entire alignment with each branch group having its own ω parameter, using $C\times \text{nucleotide}(t_{b})$ for branch lengths (C is the estimated tree scaler; this simple approximation has been used successfully by us [Kosakovsky Pond and Frost 2005] and others [Yang 2000] in the past), and θ estimates from the previous step.

Step 3: Refine nuisance parameters and group-level ω: remove constraints from branch lengths and θ then perform a maximum likelihood fit under the MG-REV-CF3×4 model.

Step 4: For each site (independently), infer parameters of interest. Fix nuisance parameters at estimates from the previous step. Estimate α as tree-wide, site-specific branch-length scaler, and estimate βi for each group class. The site-model fitted here becomes the universal alternative hypothesis (most general model) for all site-level tests. The empirical validity of such estimation procedures has been discussed in Scheffler et al. (2014). As a computational shortcut, invariable sites are skipped, because maximum likelihood parameter estimates are 0 at such sites.

**Table 8.** Parameters Relevant to Contrast-FEL.

Parameter	Definition	Type
*α*	Site-specific synonymous substitution rate	Key
*β_i_*	Site-specific nonsynonymous substitution rate for branch group *i*	Key
*ω*	Ratio of nonsynonymous substitution rate to the synonymous substitution rate	Key
*B*	Number of branches in the tree	Given
*t_b_*	Branch length parameters, number estimated = *B*	Nuisance
*θ*	Nucleotide substitution rates	Nuisance
*K*	Number of branch sets	User defined
*C*	Estimated tree scaling constant	Nuisance
*N*	Number of coding nucleotide sequences	Given
*S*	Number of codons within a sequence	Given

Note.—Parameter type is based on whether it is a key estimated parameter, a nuisance estimated parameter, a user-defined parameter, or a property of the input data set.

### Hypothesis Testing

Depending on the value of *K* and whether or not the background set is present, different testing procedures will be carried out at each site. All tests are LRTs, using the assumed asymptotic distribution of $\chi_{d}^{2}$ to test significance. The degrees of freedom parameter, *d*, varies from test to test.

1. *K* = 2. The single null hypothesis, $\beta_{1}=\beta_{2}$, is tested against the universal alternative with *d* = 1.
2. *K* > 2, no background. An omnibus test using the null $\beta_{1}=\beta_{2}=\cdots \;\beta_{K}$, versus the universal alternative, with d=K−1. In addition, all pairs of groups are tested for equality of rates using the null $\beta_{i}=\beta_{j}$ for 1≤i<j≤K with *d* = 1, resulting in K(K−1)/2 tests.
3. *K* > 2, with background. Without loss of generality, assume that the background is designated as group *K*. An omnibus test compares the null $\beta_{1}=\beta_{2}=\cdots \;\beta_{K}-1$, against the universal alternative, with d=K−2. In addition, if *K* > 3, all pairs of groups are tested for equality of rates using the null $\beta_{i}=\beta_{j}$ for 1≤i<j≤K−1 with *d* = 1, resulting in (K−1)(K−2)/2 tests.

When multiple hypotheses are tested at each site, the corresponding *P*-values are corrected to maintain nominal FWER using the Holm–Bonferroni (Holm 1979) procedure.

### Permutation Tests

As an option, it is possible to perform branch set permutation tests at each site where some of the LRTs from the previous section are significant at a given level (e.g., p≤0.05) to assess whether or not the differences detected across groups are due to “outlier” effects. To do so, we randomly shuffle branch assignments to sets (maintaining the number and size of the sets) and perform the complete LRT procedure described above for each permuted branch set, up to 20 times. If we find the LRT as high or higher as observed on the original partition for *any* of the tests, at iteration *j*, we report permutation *P*-value of 1/j (and stop the process); otherwise, we report a permutation *P*-value of 0.05.

### Final Reports

For each site that is not invariable, Contrast-FEL outputs Holm–Bonferroni corrected *P*-values for each of the conducted tests, (if selected) the overall permutation *P*-value, and *q*-values (FDRs) computed from the omnibus test *P*-values using the Benjamini and Hochberg (1995) procedure.

### Simulated Data

To evaluate Contrast-FEL statistical and predictive performance, we simulated 3,706 data sets with varying numbers of sequences, gene-size variable lengths, and other settings designed to cover a range of relevant parameter values. In total, there were 1,594,400 codon sites across all alignments.

- *Tree topologies*. Two tree topology schemes were used for the simulated. First, we simulated data along a single, empirical tree topology with reported branch lengths from Yokoyama et al. (2008) containing *N* = 31 taxa, chosen to represent a somewhat typical use case for these types of studies. Second, we also simulated data from multiple random, balanced (maximally symmetric) and ladder-like (maximally asymmetric) topologies with *N* from 8 to 256 sequences (drawn uniformly using Latin hypercube sampling [LHC], see below). This was used in simulation analyses illustrated in figure 2*D* where the number of branches in the test/reference set needed to be variable.
- *Branch lengths*. For each parametrically generated topology, we drew the mean branch length uniformly from 0.001 to 0.25 using LHC, and then generated branch lengths from the exponential distribution with this mean.
- *Alignment length*. Integer alignment length was drawn (uniformly) from the 100 to 800 range using LHC.
- *Branch sets*. We used several simulations where the branches from the Yokoyama et al. (2008) tree were partitioned into two groups by hand. For parametrically simulated topologies, we selected the fraction of branches to belong to the “test” set from 0.01 to 0.8 using LHC, and the rest of the branches were in the “reference” set. We also simulated 458 data sets where branches were partitioned into four groups.
- *Fraction of sites with different selective regimes*. The proportion of sites in an alignment evolving under the null hypothesis ($\beta_{1}=\beta_{2}=\cdots \beta_{N}$) was drawn for each data set from the beta distribution with parameters p=7, q=1. There were also 300 strict null simulations, that is, simulations where all sites were evolving under the null hypothesis.
- *Site-to-site rate variation*. Synonymous rates were either constant across sites or varied according to the gamma distribution with parameters α=β∼max(0.1,θ), where *θ* was a draw from the normal distribution N(1,0.5). Nonsynonymous rates were drawn from the gamma distribution with parameters α=max(0.1,N(0.5,0.25)),β=1.

Latin hypercube sampling was performed over the set of four parameters: number of leaves, mean branch length, alignment length, and fraction of branches in the “test” set. To generate *S* LHC samples of parameter values, each parameter range is divided into *S* equiprobable intervals, and a joint sample of multiple parameters is chosen to ensure that every single interval for any parameter is sampled exactly once. LHC allows one to sample a broad range of parameter values with a relatively small number of samples.

### Implementation

The maximum likelihood estimation procedure consists of the following steps and is implemented in HyPhy v2.5.2 or later (Kosakovsky Pond et al. 2020). Steps 1–3 benefit from multicore processors via likelihood function parallelization, and step 4 can be distributed to multiple worker nodes via MPI, improving performance. Documentation on how to use and interpret the analyses and prepare data and trees for submission to Contrast-FEL is available and at hyphy.org. A version of Contrast-FEL will be maintained on the Datamonkey web service (datamonkey.org; Weaver et al. 2018).

### Sequence Alignments

Empirical alignments and phylogenetic trees used for analysis here can be downloaded from data.hyphy.org/web/contrast-FEL/in NEXUS format. Simulated data sets and simulation parameters can be downloaded from the same URL. Additional information is included in the README.md file.

### Computational Performance

Following the initial model fits, site-level tests are embarrassingly parallel, and can take full advantage of distributed computing resources (MPI clusters), and scale linearly in the number of unique site patterns. For example, on a MacBookPro 2019, 2.3 GHz Core i9 (I9-9880H), running OS X 10.15, with HyPhy version 2.5.15 and MPI with 12 processes (one per virtual core), the run times for the empirical data sets rounded up to the nearest minute were: 8 min for the epidermal leaf trichomes (58 sequences, 318 codons), 29 min for Rubisco (C3 vs. C4, 179 sequences, 447 codons), 66 min for HIV-1 envelope (131 sequences, 806 codons), and 72 min for HIV-1 RT (476 sequences, 335 codons).

## Supplementary Material

Supplementary data are available at *Molecular Biology and Evolution* online.

## Supplementary Material

msaa263_Supplementary_Data
