# Supplementary figures and images for: Contrast-FEL—A Test for Differences in Selective Pressures at Individual Sites among Clades and Sets of Branches
Source: Mol Biol Evol. 2020 Oct 16;38(3):1184–98. doi: 10.1093/molbev/msaa263 (PMC7947784; doi:10.1093/molbev/msaa263)

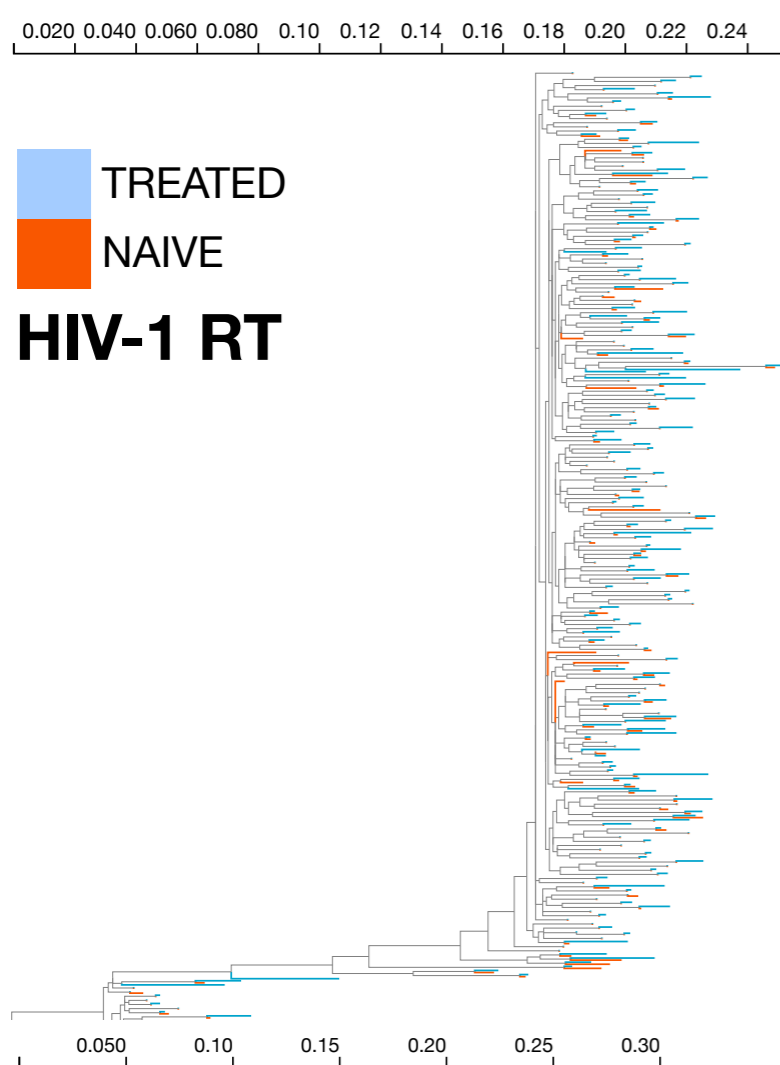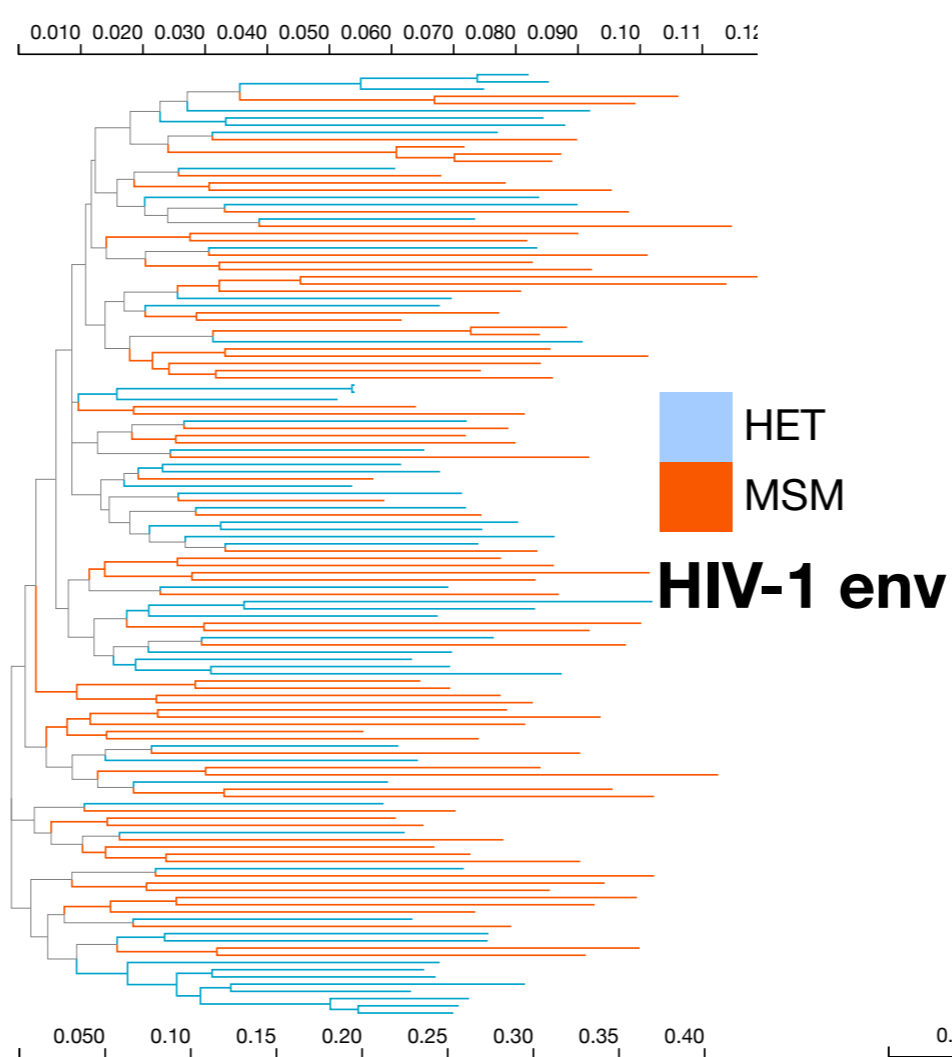

# Model misspecification

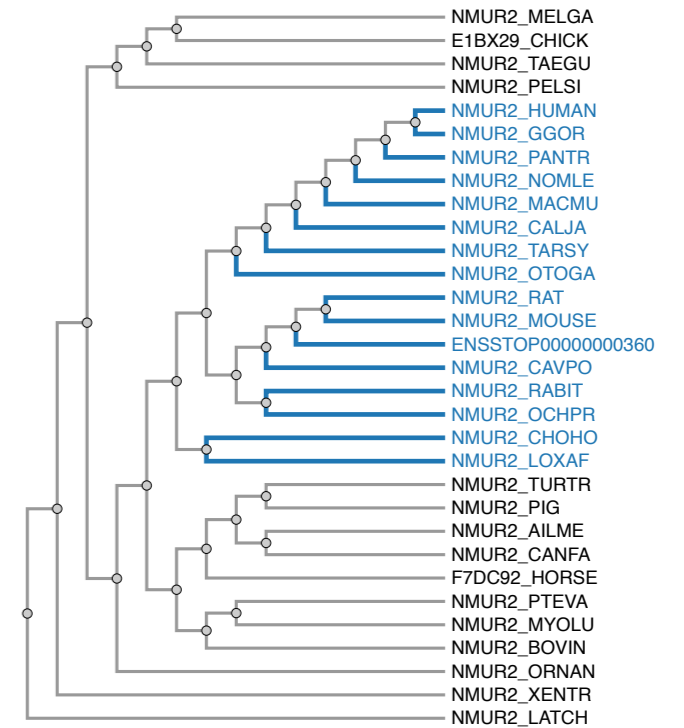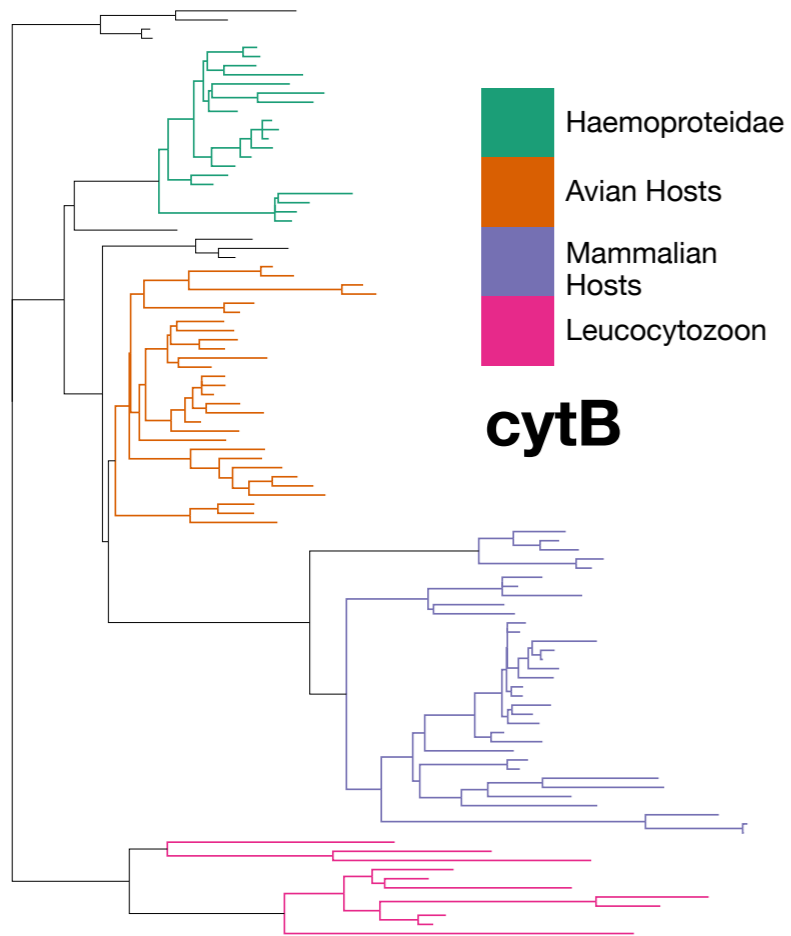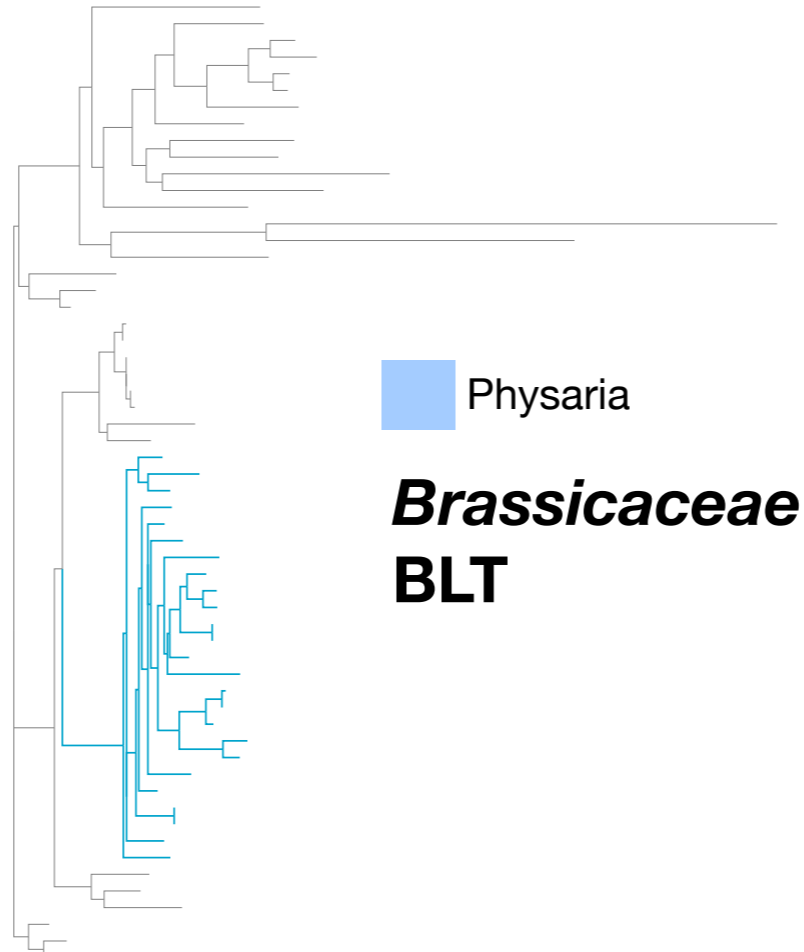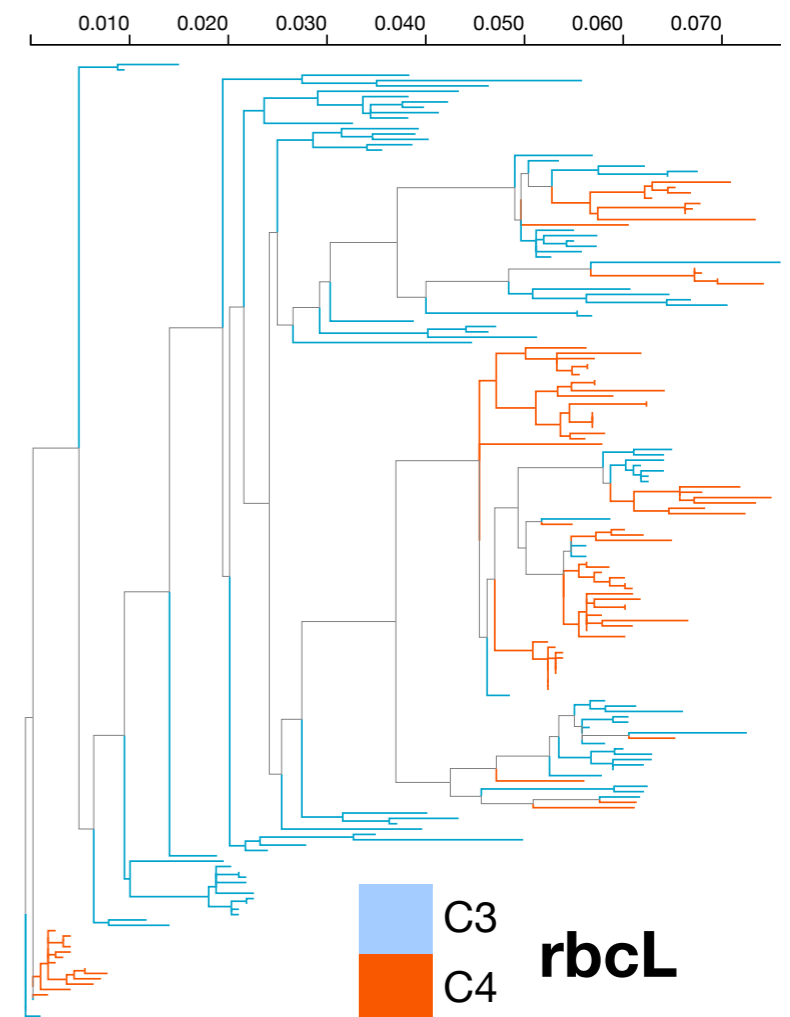

Supplement: msaa263_Supplementary_Data [file msaa263_supplementary_data.zip › FigureS1.pdf]

# Permutation

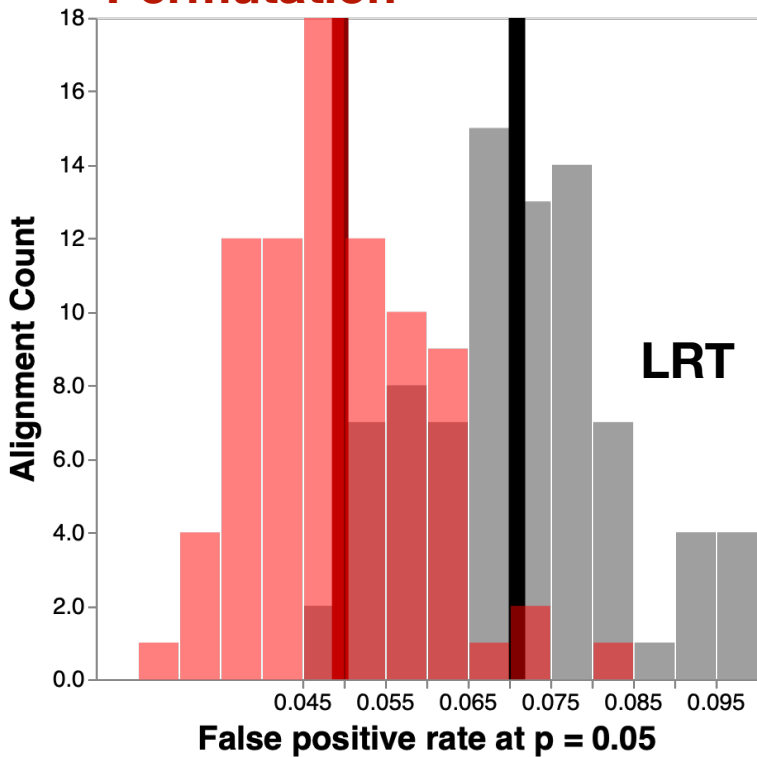

Supplement: msaa263_Supplementary_Data [file msaa263_supplementary_data.zip › FigureS2.pdf]
